# The challenges of living with bipolar disorder: a qualitative study of the implications for health care and research

**DOI:** 10.1186/s40345-018-0131-y

**Published:** 2018-11-06

**Authors:** Eva F. Maassen, Barbara J. Regeer, Eline J. Regeer, Joske F. G. Bunders, Ralph W. Kupka

**Affiliations:** 1 Athena Institute, Faculty of Earth and Life Sciences, VU University Amsterdam, Boelelaan 1085, 1081HV Amsterdam, Netherlands; 2 Altrecht Institute for Mental Health Care, Nieuwe Houtenseweg 12, 3524 SH Utrecht, Netherlands; 3 Amsterdam Public Health Research Institute, Amsterdam UMC, Vrije Universiteit Amsterdam, Psychiatry, De Boelelaan 1117, Amsterdam, Netherlands

## Abstract

**Background:**

In mental health care, clinical practice is often based on the best available research evidence. However, research findings are difficult to apply to clinical practice, resulting in an implementation gap. To bridge the gap between research and clinical practice, patients’ perspectives should be used in health care and research. This study aimed to understand the challenges people with bipolar disorder (BD) experience and examine what these challenges imply for health care and research needs.

**Methods:**

Two qualitative studies were used, one to formulate research needs and another to formulate healthcare needs. In both studies focus group discussions were conducted with patients to explore their challenges in living with BD and associated needs, focusing on the themes diagnosis, treatment and recovery.

**Results:**

Patients’ needs are clustered in ‘disorder-specific’ and ‘generic’ needs. Specific needs concern preventing late or incorrect diagnosis, support in search for individualized treatment and supporting clinical, functional, social and personal recovery. Generic needs concern health professionals, communication and the healthcare system.

**Conclusion:**

Patients with BD address disorder-specific and generic healthcare and research needs. This indicates that disorder-specific treatment guidelines address only in part the needs of patients in everyday clinical practice.

## Background

Bipolar disorder (BD) is a major mood disorder characterized by recurrent episodes of depression and (hypo)mania (Goodwin and Jamison 2007). According to the Diagnostic and Statistical Manual 5 (DSM-5), the two main subtypes are BD-I (manic episodes, often combined with depression) and BD-II (hypomanic episodes, combined with depression) (APA 2014). The estimated lifetime prevalence of BD is 1.3% in the Dutch adult population (de Graaf et al. 2012), and BD is associated with high direct (health expenditure) and indirect (e.g. unemployment) costs (Fajutrao et al. 2009; Michalak et al. 2012), making it an important public health issue. In addition to the economic impact on society, BD has a tremendous impact on patients and their caregivers (Granek et al. 2016; Rusner et al. 2009). Even between mood episodes, BD is often associated with functional impairment (Van Der Voort et al. 2015; Strejilevich et al. 2013), such as occupational or psychosocial impairment (Huxley and Baldessarini 2007; MacQueen et al. 2001; Yasuyama et al. 2017). Apart from symptomatic recovery, treatment can help to overcome these impairments and so improve the person’s quality of life (IsHak et al. 2012).

Evidence Based Medicine (EBM), introduced in the early 1990s, is a prominent paradigm in modern (mental) health care. It strives to deliver health care based on the best available research evidence, integrated with individual clinical expertise (Sackett et al. 1996). EBM was introduced as a new paradigm to *‘de*-*emphasize intuition’* and ‘*unsystematic clinical experience’* (Guyatt et al. 1992) (p. 2420). Despite its popularity *in principle* (Barratt 2008), EBM has also been criticized. One such criticism is the ignorance of patients’ preferences and healthcare needs (Bensing 2000). A second criticism relates to the difficulty of adopting evidence-based treatment options in clinical practice (Bensing 2000), due to the fact that research outcomes measured in ‘the gold standard’ randomized-controlled trials (RCTs) seldom correspond to the outcomes clinical practice seeks and are not responsive to patients’ needs (Newnham and Page 2010). Moreover, EBM provides an overview on population level instead of individual level (Darlenski et al. 2010). Thus, adopting research evidence in clinical practice entails difficulties, resulting in an implementation gap.

To bridge the gap between research and clinical practice, it is argued that patients’ perspectives should be used in both health care and research. Patients have experiential knowledge about their illness, living with it in their personal context and their care needs (Tait 2005). This is valuable for both clinical practice and research as their knowledge complements that of health professionals and researchers (Tait 2005; Broerse et al. 2010; Caron-Flinterman et al. 2005). This source of knowledge can be used in the process of translating evidence into clinical practice (Schrevel 2015). Moreover, patient participation can enhance the clinical relevance of and support for research and the outcomes in practice (Abma and Broerse 2010). Hence, it is argued that these perspectives should be explicated and integrated into clinical guidelines, clinical practice, and research (Misak 2010; Rycroft-Malone et al. 2004).

Given the advantages of including patients’ perspectives, patients are increasingly involved in healthcare services (Bagchus et al. 2014; Larsson et al. 2007), healthcare quality (e.g. guideline development) (Pittens et al. 2013) and health-related research (e.g. agenda setting, research design) (Broerse et al. 2010; Boote et al. 2010; Elberse et al. 2012; Teunissen et al. 2011). However, patients’ perspectives on health care and on research are often studied separately. We argue that to be able to provide care focused on the patients and their needs, care and research must closely interact.

We hypothesize that the challenges BD patients experience and the associated care and research needs are interwoven, and that combining them would provide a more comprehensive understanding. We hypothesize that this more comprehensive understanding would help to close the gap between clinical practice and research. For this reason, this study aims to understand the challenges people with BD experience and examine what these challenges imply for healthcare and research needs.

## Methods

To understand the challenges and needs of people with BD, we undertook two qualitative studies. The first aimed to formulate a research agenda for BD from a patient’s perspective, by gaining insights into their challenges and research needs. A second study yielded an understanding of the care needs from a patient’s perspective. In this article, the results of these two studies are combined in order to investigate the relationship between research needs and care needs. Challenges are defined as ‘difficulties patients face, due to having BD’. Care needs are defined as that what patients ‘desire to receive from healthcare services to improve overall health’ (Asadi-Lari et al. 2004) (p. 2). Research needs are defined as that what patients ‘desire to receive from research to improve overall health’.

### Study on research needs

In this study, mixed-methods were used to formulate research needs from a patient’s perspective. First six focus group discussions (FGDs) with 35 patients were conducted to formulate challenges in living with BD and hopes for the future, and to formulate research needs arising from these difficulties and aspirations. These research needs were validated in a larger sample (n = 219) by means of a questionnaire. We have reported this study in detail elsewhere (Maassen et al. 2018).

### Study on care needs

This study was part of a nationwide Dutch project to generate a practical guideline for BD: a translation of the existing clinical guideline to clinical practice, resulting in a standard of care that patients with BD could expect. The practical guideline (Netwerk Kwaliteitsontwikkeling GGZ 2017) was written by a taskforce comprising health professionals, patients. In addition to the involvement of three BD patients in the taskforce, a systematic qualitative study was conducted to gain insight into the needs of a broader group of patients.

#### Participants and data collection

To formulate the care needs of people with BD, seven FGDs were conducted, with a total of 56 participants, including patients (n = 49) and caregivers (n = 9); some participants were both patient and caregiver. The inclusion criteria for patients were having been diagnosed with BD, aged 18 years or older and euthymic at time of the FGDs. Inclusion criteria for caregivers were caring for someone with BD and aged 18 years or older. To recruit participants, a maximum variation sampling strategy was used to collect a broad range of care needs (Kuper et al. 2008). First, all outpatient clinics specialized in BD affiliated with the Dutch Foundation for Bipolar Disorder (Dutch: Kenniscentrum Bipolaire Stoornissen) were contacted by means of an announcement at regular meetings and by email if they were interested to participate. From these outpatient clinics, patients were recruited by means of flyers and posters. Second, patients were recruited at a quarterly meeting of the Dutch patient and caregiver association for bipolar disorder. The FGDs were conducted between March and May 2016.

The FGDs were designed to address challenges experienced in BD health care and areas of improvement for health care for people with BD. The FGDs were structured by means of a guide and each session was facilitated by two moderators. The leading moderator was either BJR or EFM, having both extensive experience with FGD’s from previous studies. The first FGD explored a broad range of needs. The subsequent six FGDs aimed to gain a deeper understanding of these care needs, and were structured according to the outline of the practical guideline (Netwerk Kwaliteitsontwikkeling GGZ 2017). Three chapters were of particular interest: diagnosis, treatment and recovery. These themes were discussed in the FGDs, two in each session, all themes three times in total. Moreover, questions on specific aspects of care formulated by the members of the workgroup were posed. The sessions took 90–120 min. The FGDs were audiotaped and transcribed verbatim. A summary of the FGDs was sent to the participants for a member check.

#### Data analysis

To analyze the data on challenges and needs, a framework for thematic analysis to identify, analyze and report patterns (themes) in qualitative data sets by Braun and Clarke (2006) was used. First, we familiarized ourselves with the data by carefully reading the transcripts. Second, open coding was used to derive initial codes from the data. These codes were provided to quotes that reflected a certain challenge or care need. Third, we searched for patterns within the codes reflecting challenges and within those reflecting needs. For both challenges and needs, similar or overlapping codes were clustered into themes. Subsequently, all needs were categorized as ‘specific’ or ‘generic’. The former are specific to BD and the latter are relevant for a broad range of psychiatric illnesses. Finally, a causal analysis provided a clear understanding of how challenges related to each other and how they related to the described needs.

To analyze the data on needs regarding recovery, four domains were distinguished, namely clinical, functional, social and personal recovery (Lloyd et al. 2008; van der Stel 2015). Clinical recovery refers to symptomatic remission; functional recovery concerns recovery of functioning that is impaired due to the disorder, particularly in the domain of executive functions; social recovery concerns the improvement of the patient’s position in society; personal recovery concerns the ability of the patient to give meaning to what had happened and to get a grip on their own life. The analyses were discussed between BR and EM. The qualitative software program MAX QDA 11.1.2 was used (MaxQDA).

### Ethical considerations

According to the Medical Ethical Committee of VU University Medical Center, the Medical Research Involving Human Subjects Act does not apply to the current study. All participants gave written or verbal informed consent regarding the aim of the study and for audiotaping and its use for analysis and scientific publications. Participation was voluntary and participants could withdraw from the study at any time. Anonymity was ensured.

## Results

This section is in three parts. The first presents the participants’ characteristics. The second presents the challenges BD patients face, derived from both studies, and the disorder-specific care and research needs associated with these challenges. The third part describes the generic care needs that patients formulated.

### Characteristics of the participants

In the study on care needs, 56 patients and caregivers participated. The mean age of the participants was 52 years (24–75), of whom 67.8% were women. The groups varied from four to sixteen participants, and all groups included men and women. Of all participants 87.5% was diagnosed with BD, of whom 48.9% was diagnosed with BD I. 3.5% was both caregivers and diagnosed with BD. Of 4 patients the age was missing, and from 6 patients the bipolar subtype.

### Diagnosis

**Fig. 1:**
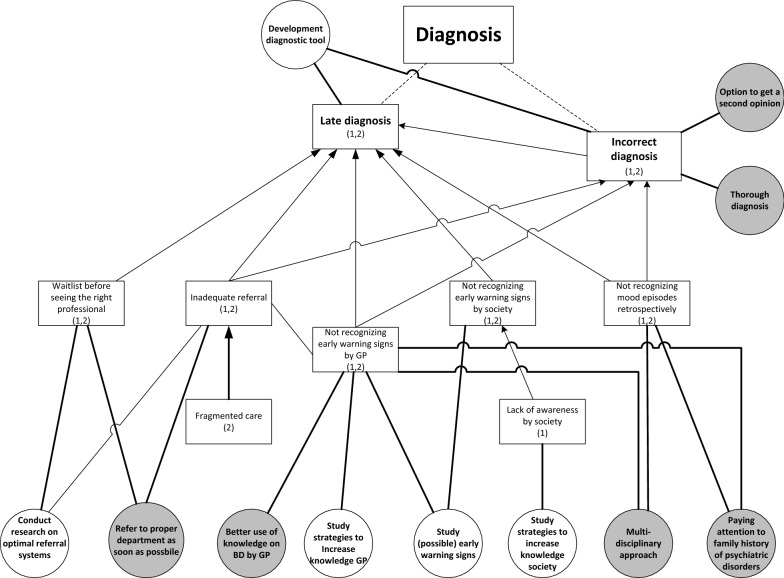
Challenges with diagnosis (squares) including relating research needs (white circles) and care needs (grey circles). (1): mentioned in study on research needs; (2): mentioned in study on care needs. Dotted lines: division of challenges into sub challenges. Arrows: causal relation between challenges

Despite the fact that participants acknowledge the inevitable diagnostic difficulties of a complex disorder like BD, in both studies they describe a range of challenges in different phases of the diagnostic process (Fig. 1). Patients explained that the general practitioner (GP) and society in general did not recognize early-warning signs and mood swings were not well interpreted, resulting in late or incorrect diagnosis. Patients formulated a need for more research on what early-warning signs could be and on how to improve GPs’ knowledge about BD. Formulated care needs were associated with GPs using this knowledge to recognize early-warning signs in individual patients. One participant explained that certain symptoms must be noticed and placed in the right context:

*I call it, ‘testing overflow of ideas’. [….] When it happens for the first time you yourself do not recognize it. Someone else close to you or the health professional, who is often not involved yet, must signal it.* (FG6)

Moreover, these challenges are associated with the need to pay attention to family history and to use a multidisciplinary approach to diagnosis to benefit from multiple perspectives. The untimely recognition of early symptoms also results in another challenge: inadequate referral to the right specialized health professional. After referral, people often face a waiting list, again causing delay in the diagnostic process. These challenges result in the need for research on optimal referral systems and the care need for timely referral. One participant described her process after the GP decided to refer her:

*But, yes, at that moment the communication wasn’t good at all. Because the general practitioner said: ‘she urgently has to be seen by someone’. Subsequently, three weeks went by, until I finally arrived at depression [department]. And at that department they said: ‘well, you are in the wrong place, you need to go to bipolar [department*]’. (FG1)

The challenge of being misdiagnosed is associated with the need to be able to ask for a second opinion and to have a timely and thorough diagnosis. On the one hand, it is important for patients that health professionals quickly understand what is going on, on the other hand that health professionals take the time to thoroughly investigate the symptoms by making several appointments.

### Treatment

**Fig. 2:**
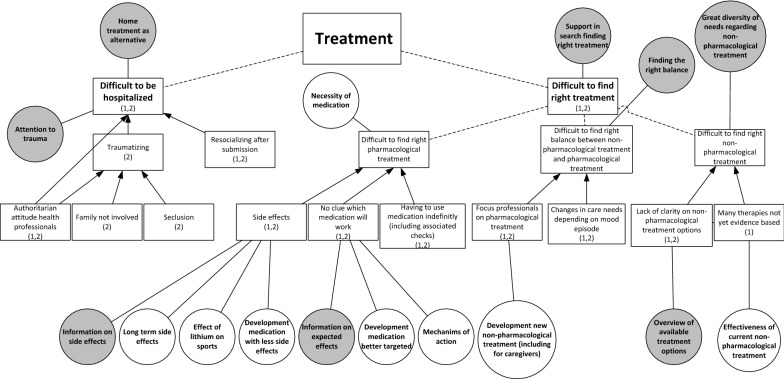
Challenges with treatment (squares) including relating research needs (white circles) and care needs (grey circles). (1): mentioned in study on research needs; (2): mentioned in study on care needs. Dotted lines: division of challenges into sub challenges. Arrows: causal relation between challenges

From both studies, two main challenges related to the treatment of BD were derived (Fig. 2). The first is finding appropriate and satisfactory treatment. Participants explained that it is difficult to find the right medication and dosage that is effective and has acceptable side-effects. One participant illustrates:

*I think, at one point, we have to choose, either overweight or depressed.* (FG1)

Some participants said that they struggle with having to use medication indefinitely, including the associated medical checks. The difficult search for the right pharmacological treatment results in the need for research on long-term side-effects, on the mechanism of action of medicine and on the development of better targeted medication with fewer adverse side-effects. In care, patients would appreciate all the known information on the side-effects and intended effects. One participant explained the importance of being properly informed about medication:

*I don’t read anything [about medication], because then I wouldn’t dare taking it. But I do think, when you explain it well, the advantages, the disadvantages, the treatment, the idea behind it, that would help a lot in compliance.* (FG1)

A second aspect is the challenge of finding non-pharmacological therapies that fit patients’ needs. They said they and the health professionals often do not know which non-pharmacological therapies are available and effective:

*But we found the carefarm ourselves*^1^ *[….]. You have to search for yourself completely. Yes, I actually hoped that that would be presented to you, like: ‘this would be something for you’.* (FG3)

Participants mentioned a variety of non-pharmacological therapies they found useful, namely cognitive behavior therapy (CBT), EMDR, running therapy, social-rhythm training, light therapy, mindfulness, psychotherapy, psychoeducation, and training in living with mood swings. They formulated the care need to receive an overview of all available treatment options in order to find a treatment best suited to their needs. They would appreciate research on the effectiveness of non-pharmacological treatments.

A third aspect within this challenge is finding the right balance between non-pharmacological and pharmacological treatment. Participants differed in their opinion about the need for medication. Whereas some participants stated that they need medication to function, others pointed out that they found non-pharmacological treatments effective, resulting in less or no medication use. They explained that the preferred balance can also change over time, depending on their mood. However, they experience a dominant focus on pharmacological treatment by the health professionals. To address this challenge, patients need support in searching for an appropriate balance.

Next to the challenge of finding appropriate and satisfactory treatment, a second treatment-related challenge is hospitalization. Participants often had a traumatic experience, due to seclusion, the authoritarian attitudes of clinical staff, and not involving their family. Patients therefore found it important to try preventing being hospitalized, for example by means of home treatment, which some participants experienced positively. Despite the challenges relating to hospitalization, participants did acknowledge that in some cases it cannot be avoided, in which case they urged for close family involvement, open communication and being treated by their own psychiatrist. Still, in the study on research needs, hospitalization did not emerge as an important research theme.

### Recovery

**Fig. 3:**
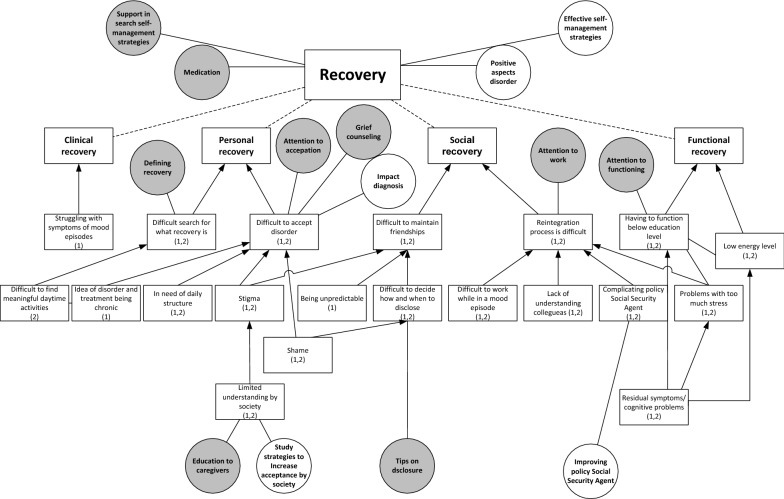
Challenges with recovery (squares) including relating research needs (white circles) and care needs (grey circles). (1): mentioned in study on research needs; (2): mentioned in study on care needs. Dotted lines: division of challenges into sub challenges. Arrows: causal relation between challenges

In both studies, participants described challenges in all four domains of recovery: clinical, functional, social and personal (Fig. 3). In relation to clinical recovery, participants struggled with the symptoms of mood episodes, the psychosis and the fear of a future episode. In contrast, some participants mentioned that they sometimes miss the hypomanic state they had experienced previously due to effective medical treatment. In the domain of functional recovery, participants contended with having to function below their educational level due to residual symptoms, such as cognitive problems, due to the importance of preventing stress in order to reduce the risk of a new episode, and because of low energy levels. This leads to the care need that health professionals should pay attention to the level of functioning of their patients.

In the domain of social recovery, participants described challenges with maintaining friendships, due to stigma, being unpredictable and with deciding when to disclose the disorder. The latter resulted in the care need for tips on disclosure. Moreover, patients experienced challenges with reintegration to work, due to colleagues’ lack of understanding, problems with functioning during an episode, the complicating policy of the (Dutch) Employee Insurance Agency^2^ in relation to the fluctuating course of BD and the negative impact of stress. These challenges are associated with the care need that health professionals should pay attention to work and the need for research on how to improve the Social Security Agency’s policy.

For their personal recovery, participants struggled with acceptance of the disorder, due to shame, stigma, having to live by structured rules and disciplines, and the chronic nature of BD. This results in care needs for grief counselling and attention to acceptance and the need for research on the impact of being diagnosed with BD. Limited understanding within society also causes problems with acceptance, corresponding with the care need for education for caregivers and for research on how to increase social acceptance. Another challenge in personal recovery was discovering what recovery means and what constitute meaningful daily activities. Patients appreciated the support of health professionals in this area. One participant described the difficult search for the meaning of recovery:

*I have been looking to recover towards the situation [before diagnosis] for a long time; that I could do what I always did and what I liked. But then I was confronted with the fact that I shouldn’t expect that to happen, or only with a lot of effort. (…) Then you start thinking, now what? A compromise. I don’t want to call that recovery, but it is a recovered, partly accepted, situation. But it is not recovery as I expected it to be.* (FG5)

In general, participants considered frequent contact with a nurse or psychiatrist supportive, to help them monitor their mood and help them find (efficient) self-management strategies. Most participants appreciated the involvement of caregivers in the treatment and contact with peers.

### Generic care needs

We have described BD-specific needs, but patients mentioned also mentioned several generic care needs. The latter are clustered into three categories. The first concerns *the health professionals*. Participants stressed the importance of a good health professional, who carefully listens, takes time, and makes them feel understood, resulting in a sense of connection. Furthermore, a good health professional treats beyond the guideline, and focuses on the needs of the individual patient. When there is no sense of connection, it should be possible to change to another health professional. The second category concerns *communication between the patient and the health professional*. Health professionals should communicate in an open, honest and clear way both in the early diagnostic phase and during treatment. Open communication facilitates individualized care, in which the patient is involved in decision making. In addition, participants wanted to be treated as a person, not as a patient, and according to a strength-based approach. The third category concerns needs at the level of *the healthcare system*. Participants struggled with the availability of the health professionals and preferred access to good care 24/7 and being able to contact their health professional quickly when necessary. Currently, according to the participants, the care system is not geared to the mood swings of BD, because patients often faced waiting lists before they could see a health professional.

*Is adequate treatment also having a number from a mental health institution you can always call when you are in need, that you can go there? And not that you can go in three weeks, but on a really short notice. So at least a phone call.* (FG3)

Participants were often frustrated by the limited collaboration between health professionals, within their own team, between departments of the organization, and between different organizations, including complementary health professionals. They would appreciate being able to merge their conventional and complementary treatment, with greater collaboration among the different health professionals. Furthermore, they would like continuity of health professionals as this improves both the diagnostic phase and treatment, and because that health professional gets to know the patient.

## Discussion

We hypothesized that research and care needs of patients are closely intertwined and that understanding these, by explicating patients’ perspectives, could contribute to closing the gap between research and care. Therefore, this study aimed to understand the challenges patients with BD face and examine what these imply for both healthcare and research. In the study on needs for research and in the study on care needs, patients formulated challenges relating to receiving the correct diagnosis, finding the right treatment, including the proper balance between non-pharmacological and pharmacological treatment, and to their individual search for clinical, functional, social and personal recovery. The formulated needs in both studies clearly reflected these challenges, leading to closely corresponding needs. Another important finding of our study is that patients not only formulate disorder-specific needs, but also many generic needs.

The needs found in our study are in line with the current literature on the needs of patients with BD, namely for more non-pharmacological treatment (Malmström et al. 2016; Nestsiarovich et al. 2017), timely recognition of early-warning signs and self-management strategies to prevent a new episode (Goossens et al. 2014), better information on treatment and treatment alternatives (Malmström et al. 2016; Neogi et al. 2016) and coping with grief (Goossens et al. 2014). Moreover, the need for frequent contact with health professionals, being listened to, receiving enough time, shared decision-making on pharmacological treatment, involving caregivers (Malmström et al. 2016; Fisher et al. 2017; Skelly et al. 2013), and the urge for better access to health care and continuity of health professionals (Nestsiarovich et al. 2017; Skelly et al. 2013) are confirmed by the literature. Our study added to this set of literature by providing insights in patients’ needs in the diagnostic process and illustrating the interrelation between research needs and care needs from a patient’s perspective.

The generic healthcare needs patients addressed in this study are clustered into three categories: *the health professional*, *communication between the patient and the health professional* and *the health system.* These categories all fit in a model of patient-centered care (PCC) by Maassen et al. (2016) In their review, patients’ perspectives on good care are compared with academic perspectives of PCC and a model of PCC is created comprising four dimensions: *patient, health professional, patient*–*professional interaction* and *healthcare organization.* All the generic needs formulated in this study fit into these four dimensions. The need to be treated as a person with strengths fits the dimension ‘patient’, and the need for a good health professional who carefully listens, takes time and makes them feel understood, resulting in a good connection with the professional, fits the dimension ‘health professional’ of this model. Furthermore, patients in this study stressed the importance of open communication in order to provide individualized care, which fits the dimension of ‘patient–professional interaction’. The urge for better access to health care, geared to patients’ mood swings and the need for better collaboration between health professionals and continuity of health professionals fits the dimension of ‘health care organization’ of the model. This study confirms the findings from the review and contributes to the literature stressing the importance of a patient-centered care approach (Mills et al. 2014; Scholl et al. 2014).

In the prevailing healthcare paradigm, EBM, the best available evidence should guide treatment of patients (Sackett et al. 1996; Darlenski et al. 2010). This evidence is translated into clinical and practical guidelines, which thus facilitate EBM and could be used as a decision-making tool in clinical practice (Skelly et al. 2013). For many psychiatric disorders, treatment is based on such *disorder*-*specific* clinical and practical guidelines. However, this disease-focused healthcare system has contributed to its fragmented nature Stange (2009) argues that this fragmented care system has expanded without the corresponding ability to integrate and personalize accordingly. We argue that acknowledging that *disorder*-*specific* clinical and practical guidelines address only parts of the care needs is of major importance, since otherwise important aspects of the patients’ needs will be ignored. Because there is an increasing acknowledgement that health care should be responsive to the needs of patients and should change from being disease-focused towards being patient-focused (Mead and Bower 2000; Sidani and Fox 2014), currently in the Netherlands generic practical guidelines are written on specific care themes (e.g. co-morbidity, side-effects, daily activity and participation). These generic practical guidelines address some of the generic needs formulated by the patients in our study. We argue that in addition to disorder-specific guidelines, these generic practical guidelines should increasingly be integrated into clinical practice, while health professionals should continuously be sensitive to other emerging needs. We believe that an integration of a disorder-centered and a patient-centered focus is essential to address all needs a patient.

### Strengths, limitations and future research

This study has several strengths. First, it contributes to the literature on the challenges and needs of patients with BD. Second, the study is conducted from a patient’s perspective. Moreover, addressing this aim by conducting two separate studies enabled us to triangulate the data.

This study also has several limitations. First, this study reflects the challenges, care needs and research needs of Dutch patient with BD and caregivers. Despite the fact that a maximum variation sampling strategy was used to derive a broad range of challenges and needs throughout the Netherlands, the Dutch setting of the study may limit the transferability to other countries. To understand the overlap and differences between countries, similar research should be conducted in other contexts. Second, given the design of the study, we could not differentiate between patients and caregivers since they participated together in the FGDs. More patients than caregivers participated in the study. For a more in-depth understanding of the challenges and needs faced by caregivers, in future research separate FGDs should be conducted. Third, due to the fixed outline of the practical guideline used to conduct the FGDs, only the healthcare needs for diagnosis, treatment and recovery of BD are studied. Despite the fact that these themes might cover a broad range of health care, it could have resulted in overlooking certain needs in related areas of well-being. Therefore, future research should focus on needs outside of these themes in order to provide a complete set of healthcare needs.

## Conclusion

Patients and their caregivers face many challenges in living with BD. Our study contributes to the literature on care and research needs from a patient perspective. Needs specific for BD are preventing late or incorrect diagnosis, support in search for individualized treatment, and supporting clinical, functional, social and personal recovery. Generic healthcare needs concern health professionals, communication and the healthcare system. This explication of both disorder-specific and generic needs indicates that clinical practice guidelines should address and integrate both in order to be responsive to the needs of patients and their caregivers.

## Footnotes

1 Care farm: farms that combine agriculture and services for people with disabilities (Iancu 2013). These farms are used as interventions in mental care throughout Europe and the USA to facilitate recovery (Iancu et al. 2014).

2 A government agency involved in the implementation of employee insurance and providing labor market and data services.
